# Severe Encephalitis Caused by Toscana Virus, Greece

**DOI:** 10.3201/eid2008.140248

**Published:** 2014-08

**Authors:** Anna Papa, Theoniki Paraforou, Ioannis Papakonstantinou, Kiriaki Pagdatoglou, Anastasia Kontana, Triantafilia Koukoubani

**Affiliations:** National Reference Centre for Arboviruses and Hemorrhagic Fever Viruses, Aristotle University of Thessaloniki, Thessaloniki, Greece (A. Papa, A. Kontana);; Trikala General Hospital, Trikala, Greece (T. Paraforou, I. Papakonstantinou, K. Pagdatoglou, T. Koukoubani)

**Keywords:** Toscana virus, phlebovirus, Greece, viruses, encephalitis, sandfly fever Naples virus

**To the Editor:** In late June 2012, a previously healthy, 49-year-old woman was admitted to the emergency department of Trikala General Hospital in Trikala, Greece, with confusion and delirium. A few hours before admission, she had had a grand mal seizure; she had experienced gastroenteritis with fever (38°C) 5 days earlier. On admission, she was intubated and transferred to the intensive care unit, where she underwent mechanical ventilation and sedation. 

The patient was a resident of Genesi village (350 m altitude), 22 km west of Trikala in the Thessaly region. She had not traveled abroad or to other area of Greece. Results of blood and cerebrospinal fluid (CSF) laboratory testing were unremarkable except slight leukocytosis (leukocytes 11,330 cells/mm^3^, 92% neutrophils) and slightly elevated serum lactate dehydrogenase level (240 U/L). Brain imaging showed edema (Technical Appendix), which resolved 48 hours after admission. The patient was awakened on day 3 of hospitalization and extubated on day 4. Treatment included anticonvulsants, mannitol, antimicrobial drugs (vancomycin and ceftriaxone), acyclovir, and corticosteroids. The patient fully recovered and was discharged from the hospital on day 12 with short-term antiepileptic medication.

Because West Nile virus (WNV) infections emerged in 2010 in Greece and outbreaks have recurred (*1*), serum and CSF samples from the patient were sent for testing to the National Reference Centre for Arboviruses. Antibodies against WNV were not detected. Reverse transcription nested PCR was conducted by using generic primers for flaviviruses, enteroviruses, and phleboviruses. PCR for phleboviruses (*2*) resulted in a PCR product of the expected size, and the sequence was most closely related to those of isolates belonging to the *Sandfly fever Naples virus* (SFNV) species (Figure). The sequence also had the highest homology (85%) with a Toscana virus (TOSV) strain belonging to lineage C that had been obtained from a patient with central nervous system infection in Croatia in 2008 (*3*). The TOSV sequence derived from the patient in this report was submitted to GenBank (accession no. KJ418710). On the basis of a partial sequence comparison (202 nt in the polymerase gene), we found that TOSV lineage C differs from lineages A and B by 29% and 30%, respectively.

**Figure:**
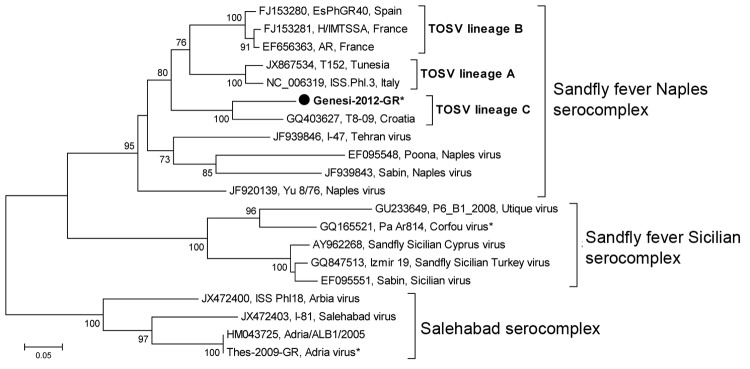
Neighboring-joining tree constructed on the basis of a 202-bp fragment of the large RNA segment of sandfly-borne phleboviruses. Black circle indicates Toscana virus strain detected in this study in a patient in Greece; asterisks (*) indicate phleboviruses detected in Greece. The percentages of replicate trees in which the associated taxa clustered together in the bootstrap test (1,000 replicates) are shown next to the branches. Evolutionary analyses were conducted in MEGA5 (http://www.megasoftware.net). Scale bar indicates substitutions per nucleotide position.

Forty-one days after symptom onset, a second serum sample was taken from the patient and tested in parallel with the first serum sample by indirect immunofluorescence to detect IgM and IgG antibodies against 4 phleboviruses: TOSV, SFNV, sandfly fever Sicilian virus (SFSV), and Cyprus virus (Sandfly Fever Virus Mosaic 1; Euroimmun, Lübeck, Germany). The first sample yielded negative results, but the follow-up sample showed IgM and IgG against TOSV and SFNV (both belonging to SFNV serocomplex). Neutralization testing to differentiate TOSV and SFNV was not performed because PCR and sequencing confirmed the TOSV infection.

Sandfly-borne phleboviruses (family *Bunyaviridae*) are endemic in Mediterranean countries, and at least 3 serotypes are associated with disease in humans: TOSV, SFNV, and SFSV. Among these, TOSV is associated with neurotropism, a major cause of meningitis and encephalitis in the Mediterranean region (*4*). Recent studies in Greece showed that the seroprevalence of TOSV (and antigenically related viruses) ranges from 0% to 60%; the higher levels are found in the islands and the coastal regions (*5*–*7*).

A study conducted in 2 Greek islands (Lefkas and Corfu, where Corfu virus was isolated) showed that the sandfly species with the widest distribution was *Phlebotomus neglectus* (31.2%), followed by *P. similis* (25.1%) and *P. tobbi* (15.3%) (*8*). In Thessaly region, where the case we report occurred, a faunistic study of sandflies showed that *P. perfiliewi* and *P. papatasi* (known vectors of TOSV) accounted for 83.4% and 3.93%, respectively, of the sandflies collected (*9*). Another phlebovirus, Adria virus (belonging to the Salehabad serocomplex), which was initially detected in sandflies collected in Albania, was detected in a febrile child with seizure in Thessaloniki in northern Greece (*10*). Concerning TOSV, however, although seroconversion has been previously observed in patients in Greece, RNA has not been detected.

For this patient, TOSV was detected by using phlebovirus generic primers. The TOSV sequence found in Greece differs greatly from other TOSV sequences, even from the genetically closer Croatian TOSV sequence (15%). To avoid false-negative results, the high genetic diversity among TOSV strains must be taken into consideration when using TOSV-specific primers.

In conclusion, a novel variant of TOSV has been detected in Greece. Further studies are needed to obtain a whole-genome sequence of the Greek TOSV strain and to identify the vector(s) of the virus. TOSV is a highly variable neurotropic phlebovirus, a characteristic that must be taken into account by laboratory scientists. Clinicians should be aware of the possibility of phlebovirus infections in Mediterranean countries and should include these viruses in the differential diagnosis of febrile illnesses observed during the warm seasons, especially in patients who exhibit neurologic symptoms.

Technical Appendix Computed tomography scan image of the brain of a 49-year-old female patient at admission to the emergency department of Trikala General Hospital, Trikala, Greece, June 2012. A Toscana virus strain was later detected in the patient.
