# Supplementary material for: Severe Encephalitis Caused by Toscana Virus, Greece
Source: Emerg Infect Dis. 2014 Aug;20(8):1417–9. doi: 10.3201/eid2008.140248 (PMC4111170; doi:10.3201/eid2008.140248)
Supplement: Technical Appendix — Computed tomography scan image of the brain of a 49-year-old female patient at admission to the emergency department of Trikala General Hospital, Trikala, Greece, June 2012. A Toscana virus strain was later detected in the patient. [file 14-0248-Techapp-s1.pdf]

Article DOI: http://dx.doi.org/10.3201/eid2008.140248

Severe Encephalitis Caused by Toscana Virus, Greece

Technical Appendix

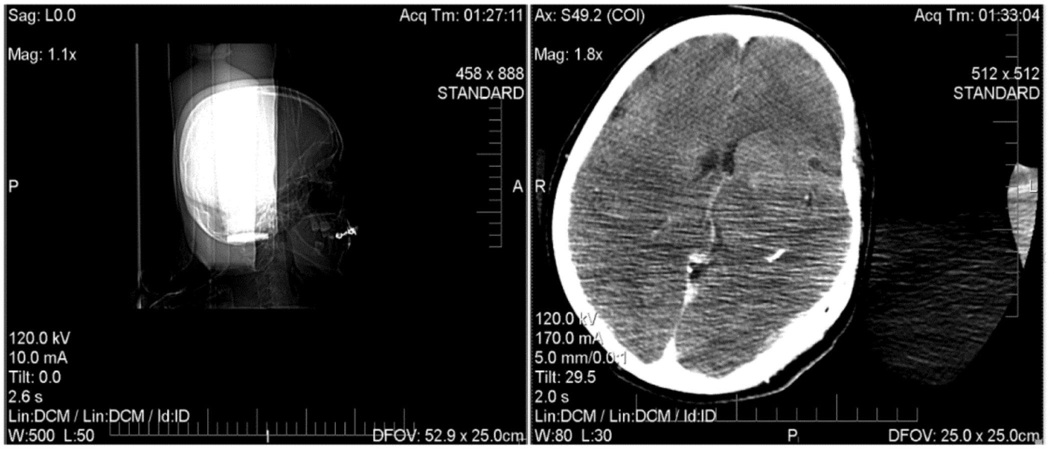

Technical Appendix Figure. Computed tomography scan image of the brain of a 49-year-old female patient at admission to the emergency department of Trikala General Hospital, Trikala, Greece, June 2012. The woman had confusion and delirium. The scan shows dilated lateral ventricles and increased attenuation of the subarachnoid spaces due to edema. A Toscana virus strain was later detected in the patient.
